# Knockout of Dopamine D3 Receptor Gene Blocked Methamphetamine-Induced Distinct Changes of Dopaminergic and Glutamatergic Synapse in the Nucleus Accumbens Shell of Mice

**DOI:** 10.3389/fncel.2022.893190

**Published:** 2022-05-30

**Authors:** Shuai Wang, Ming Li, Linlan Su, Yu Wang, Dongliang Ma, Hongyan Wang, Jie Zhu, Teng Chen

**Affiliations:** ^1^College of Forensic Medicine, Xi’an Jiaotong University Health Science Center, Xi’an, China; ^2^The Key Laboratory of Health Ministry for Forensic Science, Xi’an Jiaotong University, Xi’an, China; ^3^Programme in Neuroscience and Behavioral Disorders, Duke-NUS Medical School, Singapore, Singapore; ^4^Department of Physiology, Yong Loo Lin School of Medicine, National University of Singapore, Singapore, Singapore

**Keywords:** methamphetamine, behavioral sensitization, nucleus accumbens, ultra-structural plasticity, dopamine D3 receptor

## Abstract

Structural plasticity changes in the brain are thought to underlie, at least partially, drug-induced persistent changes in behavior. Our previous study reported that increased synaptic density in the nucleus accumbens shell (NAcsh) correlates with and may contribute to behavioral sensitization induced by methamphetamine (METH). However, the distinct changes of dopaminergic and glutamatergic synapses and the modulating effects of dopamine D3 receptor remain unclear. In the current study, we used immunohistochemistry electron-microscopy and immunofluorescence to detect the changes of dopamine D1, D2, and glutamate NR2B-positive synapses and cells in the NAcsh of METH-sensitized wild type (WT) and knockout of dopamine D3 receptor gene (D3^–/–^) mice. We found that METH induced long-term behavioral sensitization in WT mice, which was accompanied by an increased number and rate of dopamine D1 receptor-positive synapses and cells, as well as glutamate NR2B-positive synapses and cells. In contrast, the number and rate of dopamine D2 receptor-positive synapses and cells were significantly decreased in the NAcsh of METH-sensitized WT mice. D3^–/–^ mice exhibited attenuated acute locomotor responses and behavioral sensitization to METH compared with WT mice. Moreover, the knockout of dopamine D3 receptor gene inhibited METH-induced changes of dopaminergic and glutamatergic synapses in the NAcsh of METH-sensitized mice. Taken together, our results suggest that METH induced distinct changes of dopaminergic and glutamatergic synapses and cells in the NAcsh of mice, which was blocked by the knockout of dopamine D3 receptor gene, and may contribute to, at least partially, METH-induced behavior sensitization as well as the modulating effect of the dopamine D3 receptor.

## Introduction

Methamphetamine (METH) is a strong addictive and extensively abused drug, causing brain dysfunction and a series of mental symptoms (Cusick, 2017; Hanegraaf et al., 2020; Zhao et al., 2021), but the precise mechanism of METH addiction is still unclear. Previous studies focus on the mechanism underlying the transfer of casual drug administration to compulsive use of drugs (Melendez, 2011; Kawa et al., 2016; Koob, 2017). Increasing evidence suggested that drug administration induced persistent changes in the structure of brain (i.e., length and branches of dendrite, density of spine cells, and so on) (Zhang et al., 2006; Bonilla-Del Rio et al., 2021), which was thought to restructure associated neuronal connection and contribute to drug addiction (Robinson and Kolb, 2004; Torregrossa et al., 2011). Our previous studies found that increased synaptic density in the nucleus accumbens shell (NAcsh) correlates with and may contribute to behavioral sensitization induced by METH, yet the distinct changes of different types of synapses remain unclear (Zhu et al., 2012).

Nucleus accumbens (NAc), especially the NAcSh, was considered to be the key nucleus of the mesolimbic system involved in modulating drug addiction (Scofield et al., 2016). The principal cell type in the NAc is the GABAergic neurons, which express dopamine and glutamate receptors (Gerfen and Surmeier, 2011; Scofield et al., 2016). The NAcSh mainly receives glutamatergic projections from the prefrontal cortex (Ma et al., 2014), basolateral amygdala (Lee et al., 2013), and paraventricular thalamus (Pinto et al., 2003), and dopaminergic projections from the ventral tegmental area (VTA) (Beckstead et al., 1979). Meanwhile, the NAcSh projects GABAergic projections into VTA (Watabe-Uchida et al., 2012). Thus, the integration of dopaminergic and glutamatergic projections was thought to be the neuronal basis of NAc-modulate addictive behaviors (Gardner, 2011; Scofield et al., 2016). Previous studies also revealed important roles of dopaminergic and glutamatergic pathways in modulating drug addiction and associated structural plasticity (Heidbreder et al., 2005; Fenu et al., 2006; Schumann and Yaka, 2009; Shen et al., 2011; Zhang et al., 2017; Tu et al., 2019). For instance, microinjection of dopamine D1 receptor antagonist in the NAc inhibited the acquisition of morphine-induced conditioned place preference (CPP) in rats (Fenu et al., 2006), and dopamine D2 receptor antagonist inhibited the formation and expression of METH-induced behavior-sensitization in mice (Jing et al., 2018). Dopamine D1 and D2 receptors and their respective downstream molecules regulated METH-induced structural changes (length and branches of dendrite, density of spine cells) in the NAc of mice (Tu et al., 2019). Additionally, ifenprodil, a selective antagonist of NR2B, was also used to reverse heroin-induced increases of spine density in the NAc of rats (Shen et al., 2011). However, the distinct changes of dopaminergic and glutamatergic synapses in the NAcsh of METH-sensitized mice and the underlying mechanism remain unclear.

The dopamine D3 receptor is highly expressed in the NAc (Levesque et al., 1992; Leggio et al., 2016) and plays an important role in the modulation of drug addiction (Heidbreder et al., 2005; Zhang et al., 2017). Dopamine D3 receptor mediated signaling pathways regulate cocaine-induced synaptic structural modification, while dopamine D3 receptor antagonists inhibited cocaine-induced relapse (Ashby et al., 2015; Zhang et al., 2017). Our previous studies found that knockout of dopamine D3 receptor gene (D3^–/–^) mice exhibited attenuated behavioral sensitization to METH (Zhu et al., 2012; Chen et al., 2018). Unexpectedly, the knockout of dopamine D3 receptor gene did not affect the increase of synaptic density in the NAcsh of METH-sensitized mice (Zhu et al., 2012). Although the dopamine D3 receptor is not essential in modulating synaptic density in the NAcsh of METH-sensitized mice, whether the dopamine D3 receptor may affect specific synapses with dopaminergic or glutamatergic afferents remains unknown.

In the current study, we detected the structural plasticity of dopamine D1, D2, and glutamate NR2B-positive synapses in the NAcsh of METH-sensitized wild-type (WT) and D3^–/–^ mice. We also evaluated the number and rate of dopamine D1, D2, and glutamate NR2B-positive cells (D1R-positive cells, D2R-positive cells, and NR2B-positive cells). Our results revealed that METH induced distinct changes of dopaminergic and glutamatergic synapses in the NAcsh of mice, which may, at least partially, contribute to METH-induced behavior sensitization as well as the modulating effect of the dopamine D3 receptor.

## Materials and Methods

### Animals

We obtained WT and D3^–/–^ mice generated by Professor Xu (Department of Anesthesia and Critical Care, The University of Chicago). D3^–/–^ mice have been previously described (Xu et al., 1997; Zhu et al., 2012; Wang et al., 2020). Briefly, the mice were back-crossed from the 129Sv/C57BL6J genetic background to the C57BL6J background for three generations. Homozygous mutant and WT littermates were produced by crossing D3 receptor heterozygous mutant mice and were genotyped by polymerase chain reaction (PCR). And we would identify the genotypes of the mice before we performed the experiment. Mice were housed under a 12-h light/dark cycle and were housed in groups of three or four with food and water provided *ad libitum*. Both temperature and humidity of the housing room were controlled (23–25°C and 50 ± 5%). A total of 38 D3^–/–^ and WT male mice (8–12 weeks, 25–30 g) were used in the current study. All experiments were approved and performed in accordance with the guidelines of the Institutional Animal Care Committee of Xi’an Jiaotong University for the Care and Use of Laboratory Animals. All efforts were made to minimize the number of animals used and distress to the animals.

### Drugs

Methamphetamine hydrochloride was purchased from the National Institute for the Control of Pharmaceutical and Biological Products (Beijing, China). The METH dose was 2 mg/kg, which was a proper dosage in our previous research (Zhu et al., 2012; Zhao et al., 2014).

### Behavioral Experiment

**FIGURE 1:**
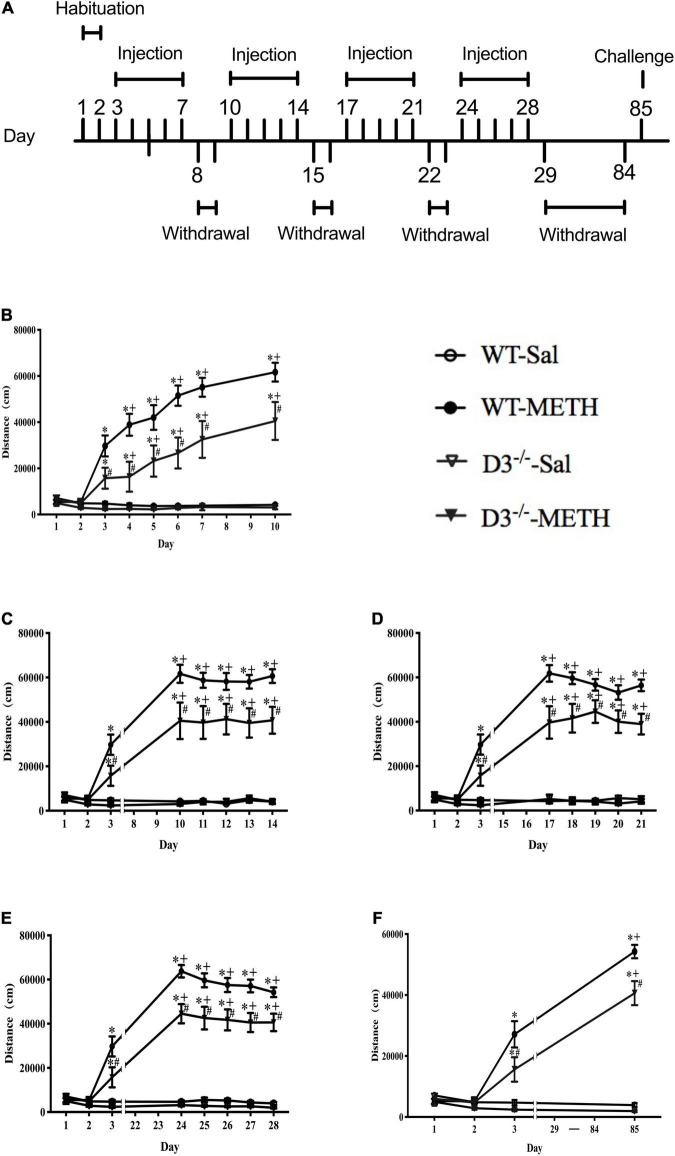
Knockout of dopamine D3 receptor gene attenuated METH-induced long-term behavioral sensitization. **(A)** Administration schedules. Days 1–2: mice were injected i.p. in volumes of 10 ml/kg once daily with saline and their horizontal locomotor activities were measured for 60 min after the injections to habituate to the open field and drug treatment. Days 3–7, days 10–14, days 17–21, days 24–28, and day 85: for the 2 mg/kg METH and saline groups, mice were given once-daily injections of either METH or saline, respectively, and their horizontal locomotor activities were determined for 60 min after the injections. Days 8–9, days 15–16, days 22–23, and days 29–84: mice were left undisturbed. **(B–F)** The locomotor activities of mice after i.p. injection of 2 mg/kg METH or saline on days 1–10 **(B)**, days 11–14 **(C)**, days 17–21 **(D)**, days 24–28 **(E)**, and day 85 **(F)**. Values are presented as mean ±SEM. *P < 0.05 compared with the same genotype in saline group; ^#^*P* < 0.05 compared with same-dose WT mice; ^+^*P* < 0.05 compared with day 3. WT, wild type.

Each mouse was handled for 5 min once daily for 7 consecutive days before all treatments were carried out to adapt the environment and the experimenter. After that, all mice were habituated to the saline intraperitoneal (i.p.) injection and chambers (43 cm × 43 cm × 43 cm, metal boxes with white bottom and black walls) for 2 days (days 1–2). All groups of mice were injected i.p. once daily with saline (0.9%, 10 ml/kg) on days 1–2 and their horizontal locomotor activities were determined by a Smart Video Tracking System (Version 2.5, Panlab Technology for Bioresearch, Spain) for 60 min after the injections. WT and D3^–/–^ mice were then divided into two groups, respectively; one group was given a dose of 2 mg/kg of METH i.p. injections (including 8 D3^–/–^ mice and 13 WT mice), and the other group was given the same number of saline i.p. injections (including 7 D3^–/–^ mice and 10 WT mice). The behavioral model was referred to previous studies (Zhu et al., 2012). Briefly, mice were given a daily injection of METH or saline for 5 consecutive days (days 3–7), followed by a withdrawal period of 2 days (days 8–9), and this cycle was repeated four times. Then, all the mice were given an 8-week withdrawal period (days 29–84) to allow potential structural changes to occur (Kolb et al., 2003; Thompson et al., 2004; Chen et al., 2008; Zhu et al., 2012). On day 85, mice were given a challenge injection of METH or saline. Horizontal locomotor activities of mice were determined on all drug treatment days for 60 min after the injections. The regimen of behavioral sensitization is shown in Figure 1A.

### Brain Sample Preparation for Electron Microscopy

Twenty-four hours after the challenge injection of METH or saline, three mice from each group were deeply anesthetized with sodium pentobarbital solution (100 mg/kg) and perfused transcardially with 500 ml of 0.1 M phosphate buffer (PB; pH 7.4) that contained 4% (w/v) paraformaldehyde and 0.1% (w/v) glutaraldehyde to prepare samples for electron microscopy (EM). The brain tissues were then cut into coronal sections (80–100 μm) through the NAc with vibratome (Leica, Germany). Subsequently, the sections were placed in 0.05 M PB containing 25% (w/v) sucrose and 10% (v/v) glycerol for 1 h and then freeze-thawed with liquid nitrogen. The sections were then incubated at room temperature with 50 mM Tris–HCl-buffered saline (TBS; pH 7.4) containing 20% (v/v) normal bovine serum for 1 h and processed for immunolabeling for dopamine D1 receptor, dopamine D2 receptor, or glutamate NR2B receptor. Briefly, the sections were incubated at room temperature for 24 h with a rabbit anti-dopamine receptor D1 (1:50, Abcam, Cat#ab20066, RRID:AB_445306), a mouse anti-dopamine receptor D2 (1:100, Santa Cruz Biotechnology, Cat#sc-5303, RRID:AB_668816), and a rabbit anti-NR2B (1:50, Abcam, Cat#ab65783, RRID:AB_1658870), respectively. After washing with TBS, the sections were incubated at 4°C for 24 h with a mixture of 1:100-diluted 1.4 nm gold particle conjugated goat antibody to rabbit IgG or mouse IgG (Nanoprobes, Cat#2001, RRID:AB_2877644; Nanoprobes, Cat#2003, RRID:AB_2687591). The sections were then postfixed with 1% (w/v) glutaraldehyde in 0.1 M PB (pH 7.4) for 10 min and washed in distilled water. After silver enhancement in the dark with the Nanoprobes lot 05D692, the sections were placed in 0.1 M PB (pH 7.4) containing 1% (w/v) OsO4 for 40 min and then counterstained with 1% (w/v) uranyl acetate in 70% ethanol for 40 min. After dehydration, the sections were mounted on silicon-coated glass slides and embedded in a fixing agent (SPI-Chem Fixatives). Once the resin had polymerized, small pieces containing the NAcsh (Paxinos et al., 2001) were cut out from the flat-embedded sections and selected tissue pieces were cut into 60 nm thick sections on an ultramicrotome (Leica, Germany). The ultrathin sections were mounted on single-slot grids coated with piloform membrane and examined with an electron microscope (Nippon Electronics 1400). Three ultrathin sections of each mouse were randomly selected for photography, and 4–6 images of each ultrathin section were taken for statistical analysis.

### Immunofluorescence

The preparation of brain samples for immunofluorescence was similar with EM. Briefly, after being perfused transcardially with saline and 4% paraformaldehyde (pH 7.4), the brain samples were post-fixed with 4% paraformaldehyde for 4 h. The brain samples were dehydrated overnight with 30% sucrose solution and embedded with optimal cutting temperature compound and frozen with liquid nitrogen. Then, the coronal brain sections (10 μm) through the NAc level were prepared with freezing microtome (Leica CM1850). After being blocked with goat serum for 30 min at room temperature, the sections were incubated with a rabbit anti-dopamine receptor D1 (1:100, Abcam, Cat#ab20066, RRID:AB_445306), a rabbit anti-dopamine receptor D2 (1:100, Proteintech, Cat#55084-1-AP, RRID:AB_10859941), and a rabbit anti-NR2B (1:100, Abcam, Cat#ab65783, RRID:AB_1658870) at 4°C overnight, respectively. The sections were washed with phosphate buffer saline (PBS) and then incubated for 1 h with Alexa Fluor 488 Goat Anti-Rabbit (1:500, Proteintech, Cat#SA00013-2, RRID:AB_2797132) at room temperature and again washed with PBS. Nuclei were counterstained with DAPI for 5 min. Fluorescence images at the same NAc level were captured using a fluorescence microscope (Zeiss of Germany) using a 20× objective. Images acquired randomly from each group were used to evaluate the number of D1, D2, NR2B receptors, and DAPI-positive cells, respectively. All the images used in the current study were obtained under an auto exposure mode to achieve the best observation effect.

### Statistical Analysis

All data were presented as the mean ± standard error of the mean (SEM). Two-way repeated-measures ANOVA with a *post hoc* multiple comparison test was used to analyze the effects of time, treatment, and genotype on behavioral sensitization and the difference in the entire time of every group or at each time point among different groups. Treatment and genotypes were defined as between-subject factors, and time was defined as within-subject factor. Two-way ANOVA was used to analyze the effects of METH and genotype on the number of positive synapses and cells, and LSD *post hoc* test was used to analyze the multiple comparison. The positive synapse was defined as synapses with three or more silver-intensified gold particles. The positive rate was calculated as the number of positive synapses divided by the total number of synapses in the image. The dopamine D1R, D2R, or NR2B-positive cells referred to those in which dopamine D1R, D2R, or NR2B was co-expressed with the DAPI. And the positive rate was calculated as the number of positive cells divided by the total number of DAPI in the image. Pearson Chi-square test was used to analyze the effects of METH and genotype on the rate of positive synapses and cells.

## Results

### Knockout of Dopamine D3 Receptor Gene Attenuated Methamphetamine-Induced Long-Term Behavioral Sensitization

We analyzed the effect of the dopamine D3 receptor on METH-induced behavioral sensitization in mice (Figure 1). There were no significant differences in baseline activity or time courses after saline injections between D3^–/–^ and WT mice, and saline injections did not induce significant changes in locomotor activity in both WT and D3^–/–^ mice (*n* = 10 or 7 mice each) (Figure 1). Although 2 mg/kg dose of METH injections induced behavioral sensitization in both WT and D3^–/–^ mice (Figure 1; ^+^*P* < 0.05 compared with day 3), two-way repeated-measures ANOVA revealed significant effects of treatment [*F*_(1_,_34)_ = 180.477, *P* < 0.001], genotype [*F*_(1_,_34)_ = 9.654, *P* < 0.01], and time [*F*_(22_,_748)_ = 40.600, *P* < 0.001], and the interactions of genotype and treatment [*F*_(1_,_34)_ = 7.298, *P* < 0.05], the interactions of time and treatment [*F*_(22_,_748)_ = 41.587, *P* < 0.001], the interactions of time and genotype [*F*_(22_,_748)_ = 1.767, *P* < 0.05], and interactions of time, genotype, and treatment [*F*_(22_,_748)_ = 2.365, *P* < 0.001]. Multiple comparisons for analysis of the differences among different groups at each time point suggested that D3^–/–^ mice exhibited significantly attenuated responses to METH compared with WT mice at all METH injection days (Figure 1; ^#^*P* < 0.05), including the challenge injection on day 85 and the first injection on day 3 (Figure 1F, ^#^*P* < 0.05; Figure 1B, ^#^*P* < 0.05). Therefore, METH induced long-lasting sensitization, which can persist for at least 8 weeks after the discontinuation of METH in both WT and D3^–/–^ mice (Figure 1; ^+^*P* < 0.05 compared with day 3). Importantly, the knockout of dopamine D3 receptor gene attenuated both the acute locomotor response (day 3, as shown in Figure 1) and behavioral sensitization to METH.

### Changes in Dopamine D1 Receptor-Positive Synapses and D1R-Positive Cells in the Nucleus Accumbens Shell of Methamphetamine-Sensitized Mice

**FIGURE 2:**
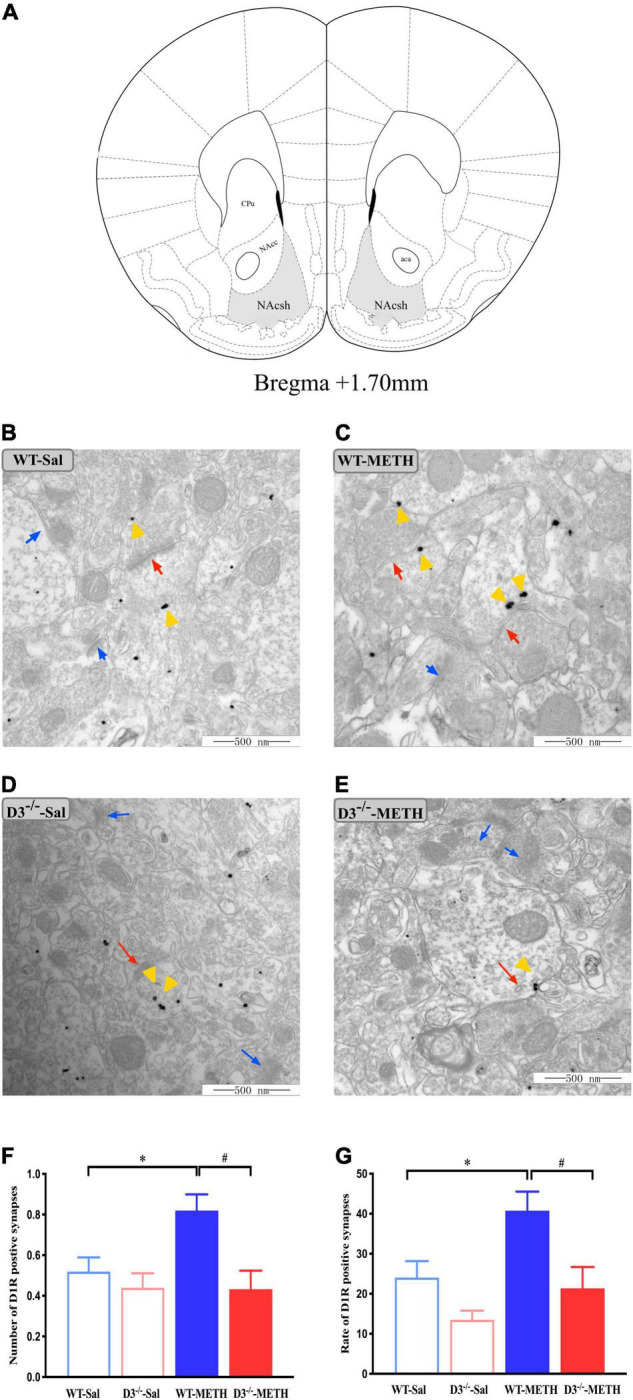
Knockout of dopamine D3 receptor gene blocked the changes of dopamine D1 receptor-positive synapses in the NAcsh of METH-sensitized mice. **(A)** Nucleus accumbens (NAc) diagram of mice. Coronal brain section representing relative sites of NAcsh (Paxinos et al., 2001) (Gray-shaded area). **(B–E)** Ultrastructure photos showed synapses in the NAcsh of mice. The red arrows indicated the dopamine D1 receptor-positive synapses, the yellow arrows indicated the silver-intensified gold particles, and the blue arrows indicated the non-positive synapses. Scale bars = 500 μm. **(F–G)** Quantification of the number **(F)** and rate **(G)** of the dopamine D1 receptor-positive synapses of the four groups of mice (*n* = 3 mice each). *P < 0.05 compared with the same genotype in saline group; ^#^*P* < 0.05 compared with same-dose WT mice. Values are presented as mean ±SEM. NAcc, nucleus accumben core; NAcsh, nucleus accumben shell; aca, anterior commissure; CPu, caudate putamen; WT, wild type.

To investigate the structural plasticity accompanied by METH-induced behavioral sensitization, we performed immunohistochemistry EM and immunofluorescence to detect the changes in dopamine D1, D2, and glutamate NR2B-positive synapses and cells in the NAcsh of METH-sensitized WT and D3^–/–^ mice. The NAcsh diagram of mice is shown in Figure 2A.

Changes in dopamine D1 receptor-positive synapses are shown in Figures 2B–G. Two-way ANOVA revealed significant effects of genotype [*F*_(1_,_185)_ = 9.029, *P* < 0.05] and the interactions of treatment and genotype [*F*_(1_,_185)_ = 3.939, *P* < 0.05], but not treatment [*F*_(1_,_185)_ = 3.562, *P* > 0.05]. Multiple comparisons found that repeated injections of 2 mg/kg dose of METH induced an increase in the number (Figure 2F; *P < 0.05 compared with the associated saline group) and rate (Figure 2G; *P < 0.05 compared with the associated saline group) of dopamine D1 receptor-positive synapses in the NAcsh of WT but not in D3^–/–^ mice (Figures 2F,G; *P* > 0.05). Moreover, the number and rate of dopamine D1 receptor-positive synapses in the NAcsh of D3^–/–^-METH group of mice were significantly lower than that of WT-METH group of mice (Figures 2F,G; ^#^*P* < 0.05). There was no significant difference between D3^–/–^ and WT saline group of mice in the number or rate of dopamine D1 receptor-positive synapses (Figures 2F,G; *P* > 0.05).

**FIGURE 3:**
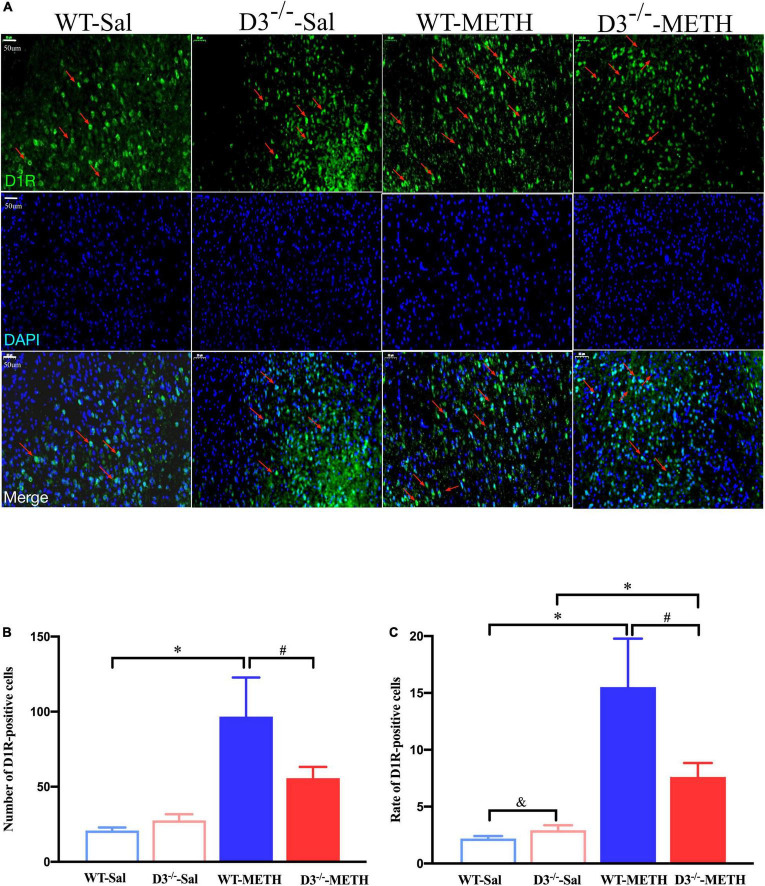
Knockout of dopamine D3 receptor gene blocked the changes of D1R-positive cells in the NAcsh of METH-sensitized mice. **(A)** Fluorescent staining of the dopamine D1 receptor in the NAcsh. The red arrows indicated the D1R-positive cells. Scale bars = 50 μm. **(B,C)** Quantification of the number **(B)** and rate **(C)** of D1R-positive cells in the NAcsh of the four groups of mice (*n* = 3 mice each). *P < 0.05 compared with the same genotype in saline group; ^#^*P* < 0.05 compared with same-dose WT mice; ^&^*P* < 0.05 compared with the different genotype in saline group. Values are presented as mean ±SEM. WT, wild type.

The results of dopamine D1 receptor-positive cells in the NAcsh of mice detected by immunofluorescence are shown in Figure 3. Two-way ANOVA revealed significant effects of treatment [*F*_(1_,_33)_ = 22.742, *P* < 0.001], and their interactions [*F*_(1_,_33)_ = 4.795, *P* < 0.05] but not genotype [*F*_(1_,_33)_ = 2.480, *P* > 0.05]. Multiple comparisons found that repeated injections of 2 mg/kg dose of METH induced an increase in the number (Figure 3B; *P < 0.05 compared with the associated saline group) and rate (Figure 3C; *P < 0.05 compared with the associated saline group) of D1R-positive cells in the NAcsh of WT mice but not in the number of D1R-positive cells in D3^–/–^ mice (Figure 3B; *P* > 0.05). Moreover, the number and rate of D1R-positive cells in the NAcsh of D3^–/–^-METH group of mice were significantly lower than that of WT-METH group of mice (Figures 3B,C; ^#^*P* < 0.05). There was no significant difference between D3^–/–^ and WT saline group of mice in the number of D1R-positive cells (Figure 3B; *P* > 0.05) and the rate of D1R-positive cells in the NAcsh of D3^–/–^-Sal group of mice was higher than that of WT-Sal group of mice (Figure 3C; ^&^*P* < 0.05).

Taken together, the knockout of dopamine D3 receptor gene inhibited the METH-induced changes in dopamine D1 receptor-positive synapses and D1R-positive cells in the NAcsh.

### Changes in Dopamine D2 Receptor-Positive Synapses and D2R-Positive Cells in the Nucleus Accumbens Shell of Methamphetamine-Sensitized Mice

**FIGURE 4:**
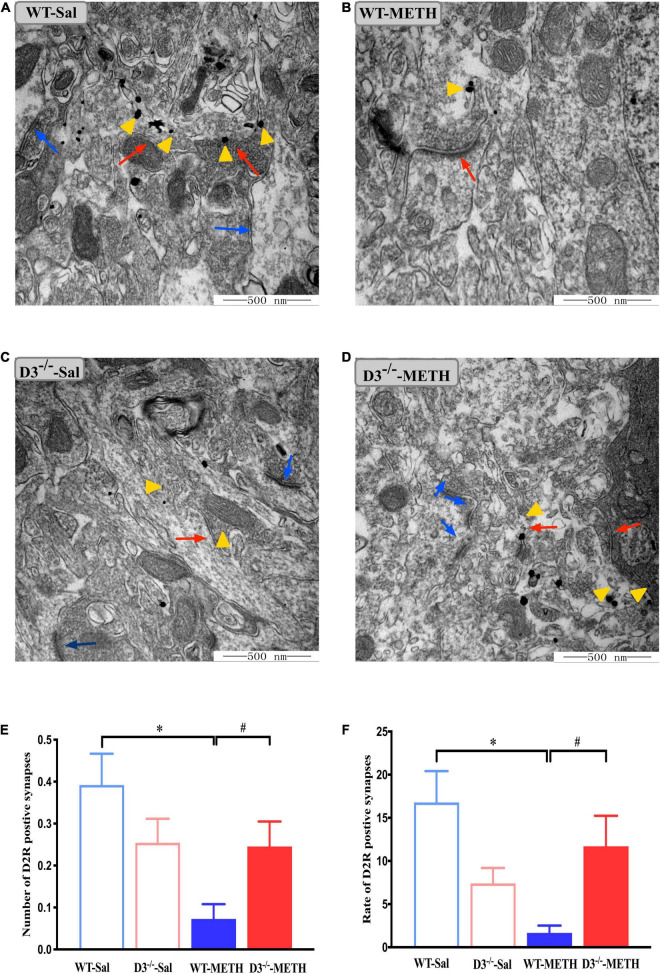
Knockout of dopamine D3 receptor gene blocked the changes of dopamine D2 receptor-positive synapses in the NAcsh of METH-sensitized mice. **(A–D)** Ultrastructure photos showed synapses in the NAcsh of mice. The red arrows indicated the dopamine D2 receptor-positive synapses, the yellow arrows indicated the silver-intensified gold particles, and the blue arrows indicated the non-positive synapses. Scale bars = 500 nm. **(E,F)** Quantification of the number **(E)** and rate **(F)** of the dopamine D2 receptor-positive synapses of the four groups of mice (*n* = 3 mice each). *P < 0.05 compared with the same genotype in saline group; ^#^*P* < 0.05 compared with same-dose WT mice. Values are presented as mean ±SEM. WT, wild type.

Next, we examined the changes in dopamine D2 receptor-positive synapses (Figure 4). Two-way ANOVA revealed significant effects of treatment [*F*_(1_,_214)_ = 8.067, *P* < 0.01], the interactions of treatment and genotype [*F*_(1_,_214)_ = 7.211, *P* < 0.001], but not genotype [*F*_(1_,_214)_ = 0.090, *P* > 0.05]. Multiple comparisons found that repeated injections of 2 mg/kg dose of METH induced a decrease in the number (Figure 4E; *P < 0.05 compared with the associated saline group) and rate (Figure 4F; *P < 0.05 compared with the associated saline group) of dopamine D2 receptor-positive synapses in the NAcsh of WT but not D3^–/–^ mice (Figures 4E,F; *P* > 0.05). Moreover, the number and rate of dopamine D2 receptor-positive synapses in the NAcsh of D3^–/–^-METH group of mice were significantly higher than that of WT-METH group of mice (Figure 4E, ^#^*P* < 0.05; Figure 4F, ^#^*P* < 0.05). There was no significant difference between D3^–/–^ and WT saline group of mice in the number or rate of dopamine D2 receptor-positive synapses (Figures 4E,F; *P* > 0.05).

**FIGURE 5:**
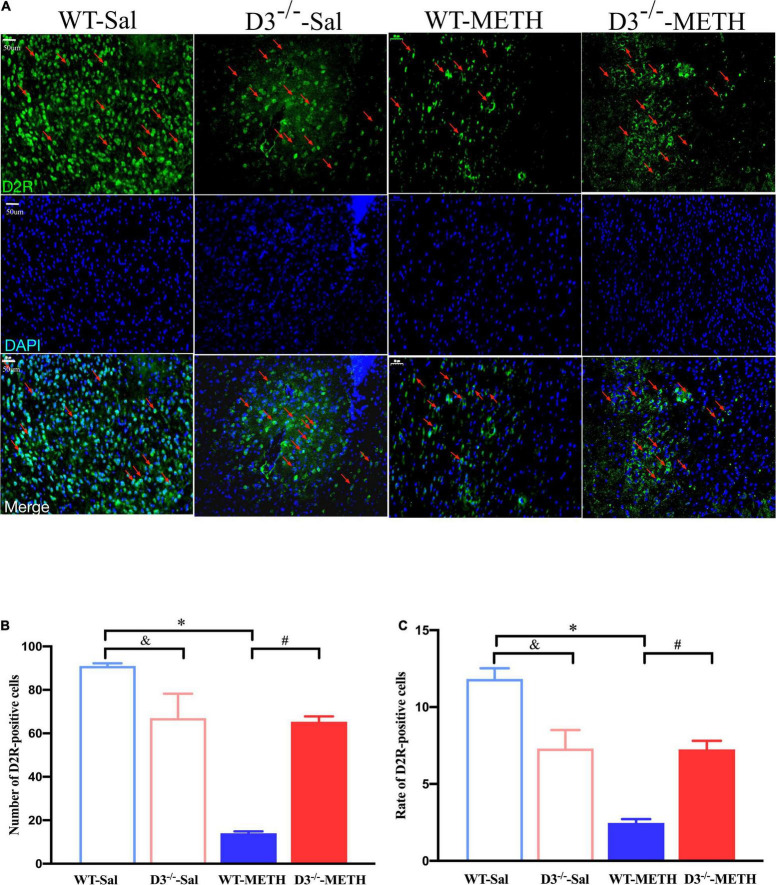
Knockout of dopamine D3 receptor gene blocked the changes of D2R-positive cells in the NAcsh of METH-sensitized mice. **(A)** Fluorescent staining of the dopamine D2 receptor in the NAcsh. The red arrows indicated the D2R-positive cells. Scale bars = 50 μm. **(B,C)** Quantification of the number **(B)** and rate **(C)** of D2R-positive cells in the NAcsh of the four groups of mice (*n* = 3 mice each). *P < 0.05 compared with the same genotype in saline group; ^#^*P* < 0.05 compared with same-dose WT mice; ^&^*P* < 0.05 compared with the different genotype in saline group. Values are presented as mean ±SEM. WT, wild type.

Immunofluorescence detected similar results of D2R-positive cells in the NAcsh of mice (Figure 5). Two-way ANOVA revealed significant effects of treatment [*F*_(1_,_18)_ = 84.709, *P* < 0.001], genotype [*F*_(1_,_18)_ = 10.227, *P* < 0.01], and their interactions [*F*_(1_,_18)_ = 77.682, *P* < 0.001]. Multiple comparisons found that repeated injections of 2 mg/kg dose of METH induced a decrease in the number (Figure 5B; *P < 0.05 compared with the associated saline group) and rate (Figure 5C; *P < 0.05 compared with the associated saline group) of D2R-positive cells in the NAcsh of WT but not in D3^–/–^ mice (Figures 5B,C; *P* > 0.05). Moreover, the number and rate of D2R-positive cells in the NAcsh of D3^–/–^-METH group of mice was significantly higher than that of WT-METH group of mice (Figures 5B,C; ^#^*P* < 0.05). The number and rate of D2R-positive cells in the NAcsh of D3^–/–^-Sal group of mice was lower than that of WT-Sal group of mice (Figures 5B,C; ^&^*P* < 0.05).

These results suggested that the knockout of dopamine D3 receptor gene inhibited the METH-induced changes in dopamine D2 receptor-positive synapses and D2R-positive cells in the NAcsh.

### Changes in Glutamate NR2B-Positive Synapses and NR2B-Positive Cells in the Nucleus Accumbens Shell of Methamphetamine-Sensitized Mice

**FIGURE 6:**
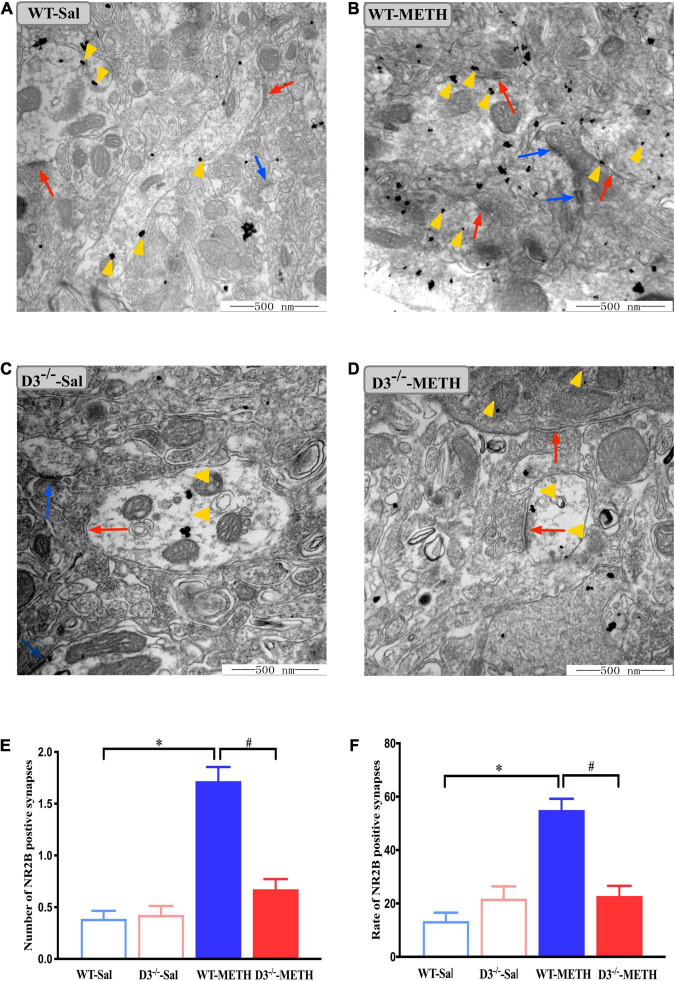
Knockout of dopamine D3 receptor gene blocked the changes of glutamate NR2B-positive synapses in the NAcsh of METH-sensitized mice. **(A–D)** Ultrastructure photos showed synapses in the NAcsh of mice. The red arrows indicated the glutamate NR2B-positive synapses, the yellow arrows indicated the silver-intensified gold particles, and the blue arrows indicated the non-positive synapses. Scale bars = 500 nm. **(E,F)** Quantification of the number **(E)** and rate **(F)** of the glutamate NR2B-positive synapses of the four groups of mice (*n* = 3 mice each). *P < 0.05 compared with the same genotype in saline group; ^#^*P* < 0.05 compared with same-dose WT mice. Values are presented as mean ±SEM. WT, wild type.

We surveyed whether the knockout of dopamine D3 receptor gene affected METH-induced changes of glutamate NR2B-positive synapses (Figure 6). Two-way ANOVA revealed significant effects of treatment [*F*_(1_,_187)_ = 64.062, *P* < 0.001], genotype [*F*_(1_,_187)_ = 26.061, *P* < 0.001], and their interactions [*F*_(1_,_187)_ = 30.165, *P* < 0.001]. Multiple comparisons found that repeated injections of 2 mg/kg dose of METH induced an increase in the number (Figure 6E; *P < 0.05 compared with the associated saline group) and rate (Figure 6F; *P < 0.05 compared with the associated saline group) of glutamate NR2B-positive synapses in the NAcsh of WT but not D3^–/–^ mice (Figures 6E,F; *P* > 0.05). Moreover, the number and rate of glutamate NR2B-positive synapses in the NAcsh of D3^–/–^-METH group of mice were significantly lower than that of WT-METH group of mice (Figure 6E, ^#^*P* < 0.05; Figure 6F, ^#^*P* < 0.05). There was no significant difference between D3^–/–^ and WT saline group of mice in the number or rate of glutamate NR2B-positive synapses (Figures 6E,F; *P* > 0.05).

**FIGURE 7:**
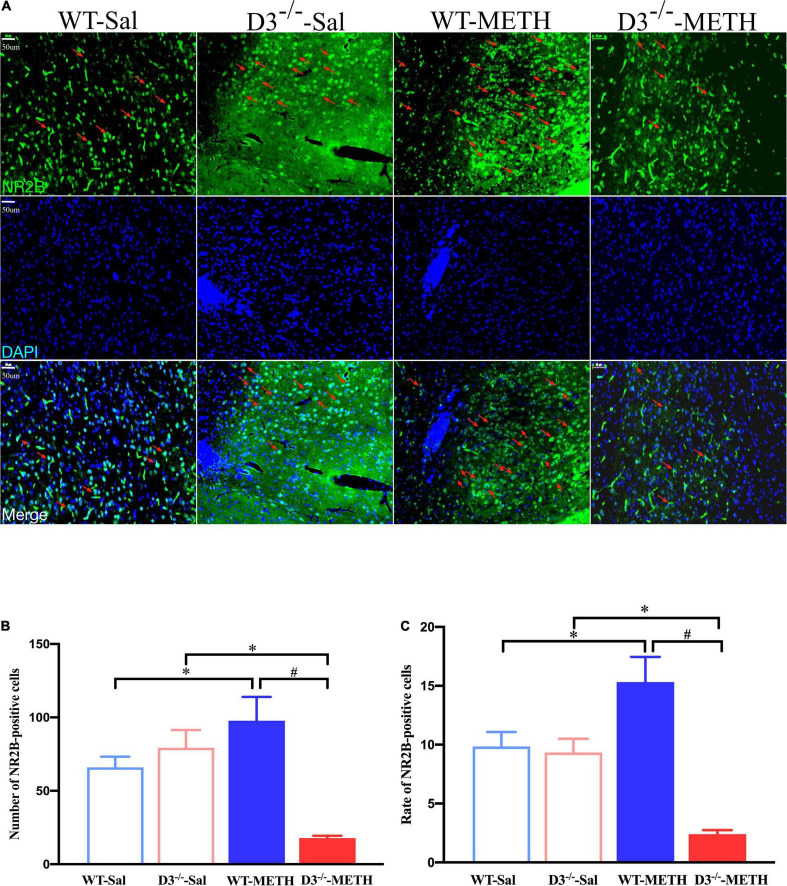
Knockout of dopamine D3 receptor gene blocked the changes of glutamate NR2B-positive cells in the NAcsh of METH-sensitized mice. **(A)** Fluorescent staining of the glutamate NR2B in the NAcsh. The red arrows indicated the glutamate NR2B-positive cells. Scale bars = 50 μm. **(B,C)** Quantification of the number **(B)** and rate **(C)** of glutamate NR2B-positive cells in the NAcsh of the four groups of mice (*n* = 3 mice each). *P < 0.05 compared with the same genotype in saline group; ^#^*P* < 0.05 compared with same-dose WT mice. Values are presented as mean ±SEM. WT, wild type.

The results of NR2B-positive cells in the NAcsh of mice detected by immunofluorescence are shown in Figure 7. Two-way ANOVA revealed significant effects of genotype [*F*_(1_,_18)_ = 11.630, *P* < 0.01], the interactions of treatment and genotype [*F*_(1_,_18)_ = 22.811, *P* < 0.001], but not treatment [*F*_(1_,_18)_ = 2.322, *P* > 0.05]. Multiple comparisons found that repeated injections of 2 mg/kg dose of METH induced an increase in the number (Figure 7B; *P < 0.05 compared with the associated saline group) and rate (Figure 7C; *P < 0.05 compared with the associated saline group) of NR2B-positive cells in the NAcsh of WT mice. In contrast, the number and rate of NR2B-positive cells in the NAcsh of D3^–/–^-METH group of mice was significantly lower than that of D3^–/–^-Sal (Figures 7B,C; *P < 0.05) and WT-METH group of mice (Figures 7B,C; ^#^*P* < 0.05). There was no significant difference between D3^–/–^ and WT saline group of mice in the number and rate of NR2B-positive cells (Figures 7B,C; *P* > 0.05).

These results suggested that the knockout of dopamine D3 receptor gene inhibited the METH-induced changes in glutamate NR2B-positive synapses in the NAcsh.

## Discussion

Drug addiction is accompanied by complex changes in the brain. Structural plasticity of the associated neuronal circuit was thought to underlie (Robinson and Kolb, 2004), at least partially, the transition from casual to compulsive patterns of drug use (Everitt and Robbins, 2005; Pierce and Vanderschuren, 2010) and drug-induced persistent changes in behavior (Robinson and Kolb, 2004). Our previous study found that increased synaptic density in the NAcsh correlates with and may contribute to behavioral sensitization induced by METH, yet the distinct changes of dopaminergic and glutamatergic synapses and the modulating effects of dopamine D3 receptor remain unclear. We thus used immunohistochemistry EM and immunofluorescence to detect the changes of dopamine D1, D2, and glutamate NR2B-positive synapses and cells in the NAcsh of METH-sensitized WT and knockout of dopamine D3 receptor gene (D3^–/–^) mice. We found that repeated METH injections induced distinct changes of dopaminergic and glutamatergic synapses and cells in the NAcsh of mice. Remarkably, the knockout of dopamine D3 receptor gene inhibited not only METH-induced long-lasting behavioral sensitization but also structural plasticity of dopaminergic and glutamatergic synapses and cells.

### Methamphetamine-Induced Changes of Dopaminergic Synaptic Structural Plasticity in the Nucleus Accumbens Shell of Mice

Nucleus accumbens is a vital component and modulator of the mesolimbic dopamine system, which was thought to play a crucial role in modulating drug addiction. The principal cell type in the NAc is GABAergic medium spiny neurons (MSNs), which comprises approximately 90–95% of the total neuronal population (Kohnomi et al., 2012; Scofield et al., 2016) and is divided into two groups based on their preferential expression of either D1-type or D2-type dopamine receptors (Gerfen and Surmeier, 2011). Moreover, there are also cholinergic interneurons in the NAc, which account for about 2% of the total NAc neurons (Hirose et al., 2021). Previous studies reported that repeated cocaine injections increased dendritic branching and spine density of MSNs in the NAcsh (Zhang et al., 2006). Moreover, deletion of dopamine D1 and D2 receptors in the NAc consistently regulated METH-induced CPP, locomotor activation, and dendritic and spine remodeling of MSNs (Tu et al., 2019). Our results showed that METH-sensitized mice were accompanied by an increased number and rate of dopamine D1 receptor-positive synapses and an increased number and rate of D1R-positive cells in the NAcsh. In contrast, the number and rate of dopamine D2 receptor-positive synapses and the number and rate of D2R-positive cells were significantly decreased in the NAcsh of METH-sensitized mice. Counterstaining of marker of distinct neurons need to be performed in further studies. Nonetheless, to the best of our knowledge, this is the first description of reverse synaptic structural plasticity of D1 or D2-positive synapses induced by repeated exposure to METH in the context of behavioral sensitization.

Previous studies showed that the D1-type MSNs in the NAc project directly to VTA dopaminergic neurons and that the D2-type MSNs in the NAc project indirectly to VTA dopaminergic neurons *via* GABAergic intermediate neurons (Russo and Nestler, 2013). Based on the specific changes of distinct synapses identified in this study, we speculated that METH induced high activity of VTA dopaminergic neurons and strengthened dopamine D1 synaptic connection in the NAcsh. These changes together enhanced activation of GABAergic neurons and GABAergic projection from NAc to VTA, and ultimately inhibited METH-induced high activity of VTA dopaminergic neurons. This negative feedback mechanism may help addicts reinstate self-stabilization and thus protect them against addiction. As for the effect of synaptic structural plasticity of dopamine D2 receptor-positive synapses and cells in the NAcsh of METH-sensitized mice, we speculate that the decreased dopamine D2 synaptic connection may lead to high activity of NAc MSNs, which then inhibited the activity of GABAergic intermediate neurons, and ultimately induced disinhibition of VTA dopaminergic neurons, and thus contribute to METH-induced behavior sensitization. Further studies are needed to examine the distinct changes of different synapses at different time points, and investigate the precise effects of distinct synaptic structural plasticity of dopamine D1 and D2 receptor-positive synapses in the NAcsh of METH-sensitized mice.

In the current study, we found that there was no significant baseline difference in dopamine D1 and D2-positive synapses between D3^–/–^ and WT mice. Unexpectedly, the rate of D1R-positive cells in D3^–/–^-Sal group was significantly higher than that of WT-Sal group, and the number of D1R-positive cells in D3^–/–^-Sal group also showed an increasing trend compared with WT-Sal group (*P* = 0.064). Moreover, the rate and number of D2R-positive cells in D3^–/–^-Sal group were both significantly lower than that in WT-Sal group. As we all know, dopamine receptors are not only expressed at synapses, but are also expressed in cell bodies and glial cell. The receptor-positive synapses detected by EM were receptors expressed at the synapse, whether the rate and number of receptor-positive cells including receptors was expressed at synapses, cell bodies, and even glial cell. This provides a possible explanation for the difference in results detected from these two methods. There are also unknown compensatory changes that occur throughout the development of knockout of dopamine D3 receptor gene mice, which may contribute to the baseline difference in the rate and number of dopamine receptor-positive cells, and may impact the current results. Future studies are needed to investigate these unresolved possibilities. Despite these limitations, our results suggested that repeated exposure to METH induced opposite synaptic structural plasticity of D1 or D2-positive synapses, which may play a distinct role in modulating METH-induced behavioral sensitization.

### Methamphetamine-Induced Changes of Glutamatergic (NR2B) Synaptic Structural Plasticity in the Nucleus Accumbens Shell of Mice

In addition to dopaminergic projection, the NAc also receives glutamatergic projection from the PFC, hippocampus, thalamus, and amygdala (Scofield et al., 2016). Indeed, a large number of studies have confirmed that the glutamatergic pathway was involved in drug-induced behavioral and structural plasticity (Ma et al., 2006; Dong et al., 2007; Shen et al., 2011). In the current study, we found that METH-sensitized mice were accompanied by an increased number and rate of glutamate NR2B-positive synapses and an increased number and rate of NR2B-positive cells in the NAcsh. The strengthened glutamate NMDA-2B synaptic connection may directly activate GABAergic neurons in NAc, which led to the hyperactivity of GABAergic pathway that originates from NAc, thus inducing inhibition (directly) or disinhibition (*via* GABAergic intermediate neurons) of VTA dopaminergic neurons (Russo and Nestler, 2013). Moreover, GABAergic intermediate neurons also exist in the NAc (Russo and Nestler, 2013; Scofield et al., 2016). Therefore, it was also possible that exposure to METH induced hyperactivity of glutamate NR2B synapses on GABAergic intermediate neurons, which then led to inhibition of GABAergic pathway that originates from NAc, and thus have an opposite effect. Whether exposure to METH induced synaptic structural plasticity on NAc GABAergic neurons or GABAergic intermediate neurons, D1 or D2-expressing MSNs, and how they regulate METH-induced behavioral sensitization remains to be further explored. Additionally, our results also showed that METH induced significant decrease in the rate and number of NR2B-positive cells in D3^–/–^ mice, but not NR2B-positive synapses, and this may due to different METH-induced changes in the expression of NR2B in synapses and cell bodies.

High doses of METH in binge-treatment regimens induce nerve terminal degeneration and neuronal apoptosis (Chapman et al., 2001; Riddle et al., 2006; Cadet and Krasnova, 2009), which may evoke morphological changes of nervous processes and spines and impact the structural plasticity of synaptic contacts. Typical neurotoxic METH regimens are in the dose range of 5–10 mg/kg given parenterally 4–10 times within 1–4 days (Seiden and Sabol, 1996; Quinton and Yamamoto, 2006; Cadet and Krasnova, 2009; Gouzoulis-Mayfrank and Daumann, 2009). Based on the available literature, 2 mg/kg METH does not induce significant neurotoxicity in the brains of the mice. Nonetheless, our previous studies have evaluated potential neurotoxic effects of METH used in the similar treatment regimen, which showed that there were no significant neurotoxic effects on the general structure of neurons, nervous processes, or spine cells in the NAcsh in either the METH or the saline groups (Zhu et al., 2012).

### Dopamine D3 Receptor Regulates Methamphetamine-Induced Behavioral Sensitization and Accompanied Synaptic Structural Plasticity in the Nucleus Accumbens Shell of Mice

Dopamine D3 receptor is more limited in distribution, mainly expressed in NAc (especially NAcsh, with a preference for the D1R-expressing MSNs) (Schwartz et al., 1998; Salles et al., 2013), hippocampus, substantia nigra, VTA, and a small amount of distribution in striatum and cerebral cortex (Bouthenet et al., 1991; Le Moine and Bloch, 1996). The dopamine D3 receptor is particularly well-positioned to influence reward and motivation and involve in drug-seeking and relapse mechanisms (Sokoloff and Le Foll, 2017). For example, previous studies found that SB-277011-A, a selective D3 receptor antagonist, could block the acquisition of heroin-induced CPP in rats (Ashby et al., 2003). YQA14, a novel dopamine D3 receptor antagonist with high affinity and selectivity for D3 receptor, has been revealed to block the expression but not the acquisition of morphine-induced CPP (Hu et al., 2013). Both SB-277011-A and YQA14 were found to inhibit methamphetamine self-administration and relapse to drug-seeking behavior in rats (Higley et al., 2011; Chen et al., 2014). Additionally, studies have shown that the dopamine D3 receptor is involved in modulating the structural plasticity of mesencephalic dopaminergic neurons (Collo et al., 2008). For instance, dopaminergic neurons from the mesencephalic of D3KO mice did not respond to cocaine, which suggested a critical role of D3R for structural plasticity induced by cocaine (Collo et al., 2012). Our previous studies found that D3^–/–^ mice exhibited attenuated behavioral sensitization to METH (Zhu et al., 2012; Chen et al., 2018). Unexpectedly, METH induced similar increases in synaptic density in the NAcsh in both WT and D3^–/–^ mice (Zhu et al., 2012). We speculated that the dopamine D3 receptor may affect specific synapses with dopaminergic or glutamatergic afferents but not overall synaptic density in the NAcsh of METH-sensitized mice. In the current study, we found that although there were no significant baseline differences in the detected synaptic structural plasticity of NAcsh between D3^–/–^ and WT mice, all METH-induced structural plasticity changes in dopaminergic and glutamatergic synapses were blocked by the knockout of dopamine D3 receptor gene.

Previous studies found that dopamine D1 receptor and D3 receptor are often co-localized on the same medium of spiny neurons (Le Moine and Bloch, 1996; Schwartz et al., 1998), and increasing evidence suggested an interaction effect of D1 and D3 receptor in modulating addictive behaviors (Marcellino et al., 2008; Galaj et al., 2016). Further studies found that D1 and D3 receptor interacted with each other by forming heteromers (Marcellino et al., 2008) or promoting changes in their ability to bind ligands and modulate intracellular signaling cascades (Galaj et al., 2016). Previous studies also revealed that dopamine D3 and D2 receptors could influence each other to form a functional D2/D3 receptor heterodimer (Scarselli et al., 2001).

Moreover, the dopamine D3 receptor was also found to modulate glutamatergic pathways either directly by interacting with the glutamate NMDA receptor in the NAc, or indirectly by controlling dopamine release from VTA neurons (Sokoloff and Le Foll, 2017). For instance, the dopamine D3 receptor could modulate morphine-induced behavioral sensitization by regulating glutamate NMDA-mediated excitatory postsynaptic currents (EPSC) in the NAc (Liu et al., 2014). Further studies are required to determine the precise mechanism of dopamine D3 receptor in modulating METH-induced structural plasticity in the NAcsh of mice. Nevertheless, our results provide new evidence to investigate the mechanism of METH-induced behavioral sensitization as well as the mechanism underlying the modulate effects of dopamine D3 receptor.

Taken together, our results suggest that METH induced distinct changes of dopaminergic and glutamatergic synapses and cells in the NAcsh of mice, which were blocked by knockout of dopamine D3 receptor gene, and may contribute to, at least partially, METH-induced behavior sensitization as well as the modulating effect of the dopamine D3 receptor.

## Data Availability Statement

The original contributions presented in the study are included in the article/supplementary material, further inquiries can be directed to the corresponding author/s.

## Ethics Statement

The animal study was reviewed and approved by Institutional Animal Care Committee of Xi’an Jiaotong University for the Care and Use of Laboratory Animals.

## Author Contributions

SW, ML, LS, and YW performed the described experiments and data analyses. JZ and TC designed the experiments and helped to analyze the results. SW, JZ, and TC wrote the manuscript. DM and HW helped to analyze the results and provided critical revisions of the manuscript. All authors contributed to the article and approved the submitted version.

## Conflict of Interest

The authors declare that the research was conducted in the absence of any commercial or financial relationships that could be construed as a potential conflict of interest.

## Publisher’s Note

All claims expressed in this article are solely those of the authors and do not necessarily represent those of their affiliated organizations, or those of the publisher, the editors and the reviewers. Any product that may be evaluated in this article, or claim that may be made by its manufacturer, is not guaranteed or endorsed by the publisher.

## Abbreviations

aca: anterior commissure
CPP: conditioned place preference
CPu: caudate putamen
D3^–/–^: knockout of dopamine D3 receptor gene
EM: electron microscopy
i.p.: intraperitoneal
METH: methamphetamine
MSNs: medium spiny neurons
NAcc: nucleus accumben core
NAcsh: nucleus accumbens shell
PB: phosphate buffer
PBS: phosphate buffer saline
TBS: Tris–HCl-buffered saline
VTA: ventral tegmental area
WT: wild-type.
